# Factors influencing the reliability of a CT angiography-based deep learning method for infarct volume estimation

**DOI:** 10.1093/bjro/tzae001

**Published:** 2024-01-05

**Authors:** Lasse Hokkinen, Teemu Mäkelä, Sauli Savolainen, Marko Kangasniemi

**Affiliations:** Radiology, HUS Medical Imaging Centre, University of Helsinki and Helsinki University Hospital, Helsinki 00290, Finland; Radiology, HUS Medical Imaging Centre, University of Helsinki and Helsinki University Hospital, Helsinki 00290, Finland; Department of Physics, University of Helsinki, Helsinki 00014, Finland; Radiology, HUS Medical Imaging Centre, University of Helsinki and Helsinki University Hospital, Helsinki 00290, Finland; Department of Physics, University of Helsinki, Helsinki 00014, Finland; Radiology, HUS Medical Imaging Centre, University of Helsinki and Helsinki University Hospital, Helsinki 00290, Finland

**Keywords:** stroke, neuroradiology, machine learning, computed tomography angiography

## Abstract

**Objectives:**

CT angiography (CTA)-based machine learning methods for infarct volume estimation have shown a tendency to overestimate infarct core and final infarct volumes (FIV). Our aim was to assess factors influencing the reliability of these methods.

**Methods:**

The effect of collateral circulation on the correlation between convolutional neural network (CNN) estimations and FIV was assessed based on the Miteff system and hypoperfusion intensity ratio (HIR) in 121 patients with anterior circulation acute ischaemic stroke using Pearson correlation coefficients and median volumes. Correlation was also assessed between successful and futile thrombectomies. The timing of individual CTAs in relation to CTP studies was analysed.

**Results:**

The strength of correlation between CNN estimated volumes and FIV did not change significantly depending on collateral status as assessed with the Miteff system or HIR, being poor to moderate (*r *=* *0.09-0.50). The strongest correlation was found in patients with futile thrombectomies (*r *=* *0.61). Median CNN estimates showed a trend for overestimation compared to FIVs. CTA was acquired in the mid arterial phase in virtually all patients (120/121).

**Conclusions:**

This study showed no effect of collateral status on the reliability of the CNN and best correlation was found in patients with futile thrombectomies. CTA timing in the mid arterial phase in virtually all patients can explain infarct volume overestimation.

**Advances in knowledge:**

CTA timing seems to be the most important factor influencing the reliability of current CTA-based machine learning methods, emphasizing the need for CTA protocol optimization for infarct core estimation.

## Background

Assessing the extent of irreversible injury, that is, the infarct core, currently plays a vital role in treatment selection in patients with acute ischaemic stroke (AIS).^1^ Infarct core volume solely or together with the volume of potentially salvageable tissue (the ischaemic penumbra) and the ratio between these two determines patient eligibility for intravenous thrombolysis or endovascular therapies.^2^^,^^3^ According to European and American guidelines, the preferred methods to determine the infarct core and penumbra are MRI diffusion-weighted-imaging (DWI) and MR or CT perfusion (CTP).^1^^,^^4^ Owing to availability issues outside larger stroke centres,^5^ there has been a search for alternative methods of determining the core volume.

CT angiography (CTA) has been found to be superior to non-contrast CT (NCCT) in determining infarct core extent, especially in the early time window.^6^ With the advent of machine learning applications in medical imaging, various automated CTA-based approaches for ischaemic tissue detection and volume estimation have been proposed.^7–11^ In our previous publications, we reported a good correlation between core estimates from a CTA-based convolutional neural network (CNN) and final infarct volumes (FIV) in patients treated with intravenous thrombolysis or supportive care, but found a poorer correlation in patients successfully treated with thrombectomy.^12^^,^^13^ This raises the question of whether the quality of collateral circulation had an effect on the reliability of CNN estimates as it has been shown previously that good collateral circulation is associated with lower ischaemic core growth and smaller 24-h ischaemic core volume in both patients with successful thrombectomy and those not treated with reperfusion therapies.^14^^,^^15^

In this study, we aimed to analyse whether differences in collateral circulation or the outcome of thrombectomy affect the reliability of CNN estimations by comparing correlations between CNN estimations and FIV between different collateral classes and successful and futile thrombectomies. The effect of CTA timing is also known to affect the reliability of core estimates.^16^ Therefore we also analysed the timing of the CTAs of our study population based on a time attenuation curve derived from patients’ respective CTP studies.

## Methods

The data that support the findings of this study are available from the corresponding author upon reasonable request. Helsinki University Hospital ethical committee approved this retrospective study (research license HUS/211/2020) and patients’ informed consent was waived.

### Study population

The clinical and imaging findings of consecutive suspected stroke cases that presented to Helsinki University Hospital between January 2018 and December 2019 were studied retrospectively. A total of 443 thrombectomies were performed during this period. Inclusion criteria for this study were: (1) admission stroke protocol imaging using fast CTA acquisition protocol and CTP, (2) anterior circulation large vessel occlusion (LVO) at CTA (distal ICA, MCA M1, MCA M2), and (3) a follow-up NCCT or an MRI study with DWI performed no later than 3 days after symptom onset. Patients with haemorrhagic transformation of infarct resulting in mass effect (*n* = 32) were excluded. This exclusion was done so that the haematomas and related oedema would not result in errors in infarct volume measurements. Patients with failed intra-arterial access (*n* = 6), a failed CTA, CTP, or follow-up imaging study (*n* = 8), or a re-thrombectomy within 1 week (*n* = 4) were also excluded. Using these criteria, 121 patients were left for analysis.

### Image acquisition and pre-processing

A majority of patients (*n* = 108) were imaged in the acute setting using a Somatom Definition Edge (Siemens Healthineers, Erlangen, Germany) 128-slice CT scanner. The CTA parameters were tube voltage 120 kVp, reference current time 150 mAs, pitch 1.3, reconstruction kernel I30f, and slice thickness/increment 0.75/0.5 mm. The iodine concentration of the contrast agent was 350 mg/mL with an amount of 50 mL and injection rate of 5 mL/s. The timing of the scan was 12 s after time to peak of the test bolus in the ascending aorta. Six patients were imaged with a 128-slice Siemens Somatom Definition Flash, three patients with a 64-slice Siemens Somatom Definition AS, two patients with a 128-slice AS+, and two patients with a 128-slice Revolution EVO (GE Healthcare, Milwaukee, WI). In 109 patients, CTP was performed before CTA acquisition and in 12 patients after the CTA. CT was used for follow-up in 111 patients and 10 patients underwent a follow-up MRI. A majority (*n* = 93) of the follow-up studies were performed using the same scanner as the CTA (Somatom Definition Edge). Ten follow-up studies were performed with a Somatom Definition Flash and eight with a Revolution EVO. Follow-up MRI studies were performed with a Siemens Magnetom Verio 3 T (*n* = 4), a Siemens Magnetom Skyra 3 T (*n* = 3), and a Siemens Magnetom Avanto 1.5 T (*n* = 3). Almost all follow-up studies were conducted 24 h (±6 h) after admission (*n* = 113). Images were anonymized and stored on a server running the Extensible Neuroimaging Archive Toolkit, version 1.1.6.^17^

All follow-up studies were evaluated for FIV by a neuroradiologist in training (LH) with 7 years of experience. The infarcted regions were segmented on follow-up CT and diffusion-weighted MRI scans using 3D Slicer Image processing and visualization platform.^18^ Collaterals were assessed visually from a maximum intensity projection of a single-phase baseline CTA by LH and assigned one of three grades: (1) poor, (2) moderate, and (3) good, using the Miteff system.^19^ All previous and future CT and MR studies, if available, were used by the radiologist to help segment the infarcts as accurately as possible. Equivocal cases were resolved in agreement with a senior neuroradiologist with 20 years of experience (MK). Image data pre-processing and 3D CNN implementation were conducted by a physicist (TM).

**Figure 1.**
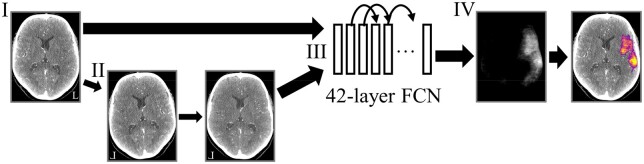
An overview of the neural network processing pipeline. (I) A CT angiography image volume is cropped to include only the cranial region and resampled to a standard isotropic resolution. (II) A left-right mirrored copy of the volume is non-rigidly registered with the original volume to incorporate local information from corresponding contralateral regions. (III) The two volumes resulting from steps I and II are used as inputs for a 42-layer deep, three-dimensional, fully convolutional network (FCN), which (IV) performs voxel-level predictions of the ischaemic area. The curved arrows indicate skip connections facilitating gradient propagation during training. A threshold of predicted probability ≥0.5 is used to produce the final segmentation. The FCN model was initially trained using data from 25 stroke and 25 control patients, with manually outlined ischaemic areas, with a Sørensen-Dice coefficient of 0.61 (95% CI: 0.52-0.67).^20^

The CNN model (Figure 1) had been previously trained and validated against expert segmentations. A detailed description of the CNN architecture can be found in a previous publication.^20^

### Study design

Lesion volumes from CNN outputs and manual segmentations of final infarcts were calculated for all lesions in the affected cerebral hemisphere. Only lesions in the affected cerebral hemisphere were selected for analysis using a volume threshold of > 0.1 mL and a probability threshold of 0.5 for lesion inclusion. False positive lesions in the contralateral hemisphere or cerebellum were excluded from the analysis. This approach was chosen because the site of arterial occlusion is readily identifiable from CTA in LVO cases.

Commercial software, Rapid CTP (iSchemaView, Menlo Park, CA), was used to calculate infarct core (cerebral blood flow <30%) and ischaemic penumbra (time-to-maximum > 6 s) volumes as well as hypoperfusion intensity ratios (HIRs) from computed tomography perfusion data. HIR values were used as a surrogate marker for collateral circulation assessment in addition to visual assessment. Based on previous studies, HIR values <0.5 and ≥0.5 were considered representative of good and poor collaterals, respectively, although lower thresholds have also been proposed in the literature.

Convolutional neural network performance was compared against manually segmented FIV, and the effect of collateral circulation was assessed based on the Miteff system and HIR-values. Thrombectomies were classified as successful or futile based on the Thrombolysis in Cerebral Infarction (TICI) classification as assessed by the interventional radiologist who performed the procedure. Thrombectomies with TICI classifications of 0, 1, or 2a were considered as futile and 2b, 2c, or 3 as successful. Comparisons between CNN estimations and Rapid CTP-derived infarct core and penumbra estimations were also made. All collateral grades were also assessed for possible differences between patients presenting <6 h or 6-24 h from last known well (LKW). LKW time was presumed to be the midpoint between going to sleep and waking up in patients presenting with wake-up stroke.

**Figure 2.**
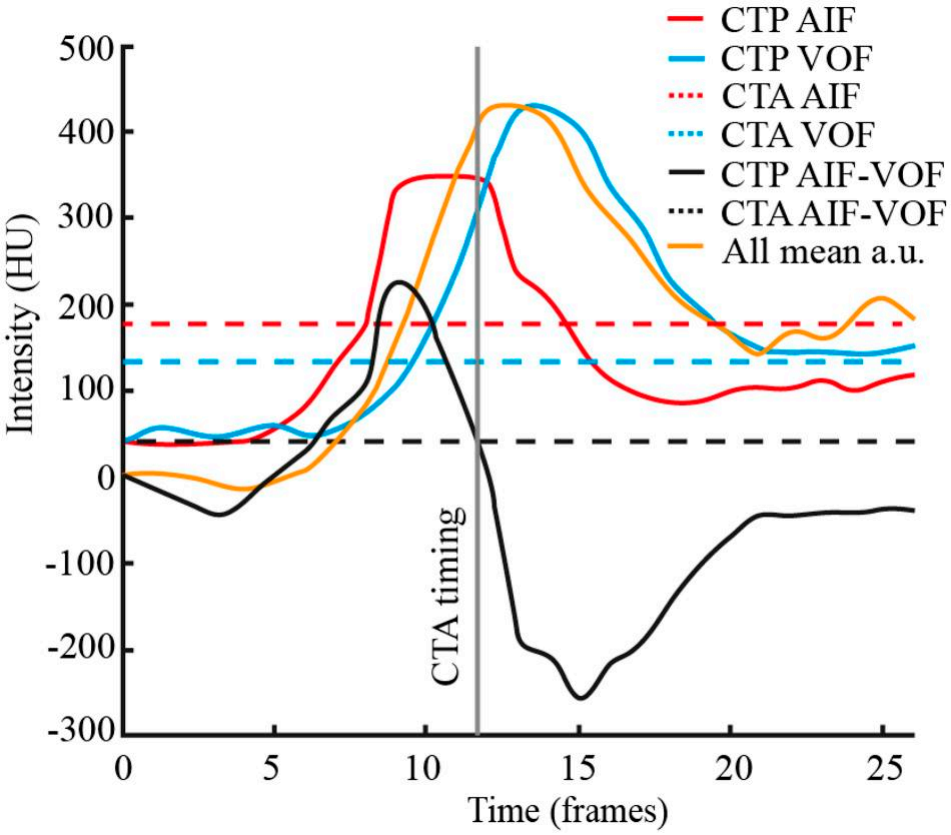
A representation of the CTA timing analysis. The orange line represents the mean HU-value across the entire field of view as a surrogate marker for whole brain attenuation. Dotted lines represent HU-values from arterial and venous regions of interest from the CTA study and are constant due to image acquisition at a single time point. Continuous lines represent HU-values from AIF and VOF of the CTP study. CTA timing was deducted from the intersection of the difference of AIF and VOF curves on CTA and CTP studies. Abbreviations: CTA = computed tomography angiography, HU = Houndsfield unit, AIF = arterial input function, VOF = venous output function, AIF-VOF = VOF subtracted from AIF.

Finally, the timing of individual single-phase CTA studies in relation to dynamic CTP data from the same patient was assessed. Each study was assigned to one of four time points on the time attenuation curve, (1) early arterial, (2) mid-arterial, (3) between mid-arterial and delayed venous phase, and (4) delayed venous phase. For this analysis, regions of interest (ROIs) were placed on either the supraclinoid ICA or ACA and superior sagittal sinus on corresponding axial slices of both CTA and CTP studies to assess Houndsfield unit (HU)-values of AIF and VOF, respectively. CTP studies were motion corrected by rigidly co-registering the subsequent timesteps with the first timestep using the BRAINSFit tool.^21^ 3D Slicer was used for ROI placement. After extracting the HU-values from the ROIs, a time attenuation curve was created for the CTP study for both AIF and VOF. A third curve representing the mean HU-value across the whole field of view was calculated as a surrogate marker for overall brain attenuation. HU-values from the corresponding CTA study were superimposed on these curves. To assess CTA timing in relation to the AIF, VOF and overall HU-curve, the HU-value of the VOF was subtracted from the AIF to create another curve. CTA timing was deducted from the intersection of the difference of between the AIF and VOF curves in the CTA and CTP studies. A representation of this analysis is shown in Figure 2.

### Statistical analysis

A linear model was fitted between CNN-derived volume outputs and manually segmented FIVs and Rapid CTP derived volumes. Pearson correlation coefficients (*r*) as well as intraclass correlation coefficients and their 95% CI were calculated to evaluate the correlation of CNN derived volumes against FIV in different subgroups (Table 2) and enable comparison with prior studies. Normality of data was assessed using the Shapiro-Wilk test. Differences in median volumes of CNN estimates as well as FIV across different collateral and TICI groups were compared using the Kruskal-Wallis test (Table 3). Statistical tests were two-sided and were considered significant with *P* < .05. Statistical analyses were performed using SPSS Statistics for Windows, version 28.0 (IBM Corp., Armonk, NY).

**Table 2.** Pearson correlation coefficients and intraclass correlation coefficients, their 95% CI and corresponding *P*-values between convolutional neural network infarct volume estimates and final infarct volumes according to collateral status assessed with Miteff scale or hypoperfusion intensity ratio and outcome of thrombectomy in all patients, patients presenting <6 h from last known well (LKW) and patients presenting 6-24 h from LKW.

	Miteff						HIR				TICI			
All patients (*n* = 121)	1 (*n* = 47)	*P*-value	2 (*n* = 42)	*P*-value	3 (*n* = 32)	*P*-value	<0.5 (*n* = 81)	*P*-value	≥0.5 (*n* = 40)	*P*-value	0, 1, or 2a (*n* = 23)	*P*-value	2b, 2c, or 3 (*n* = 98)	*P*-value
Pearson	0.32 (0.04-0.56)	.03	0.21 (−0.10 to 0.48)	.19	0.34 (−0.01 to 0.62)	.05	0.40 (0.20-0.57)	<.001	0.38 (0.07-0.62)	.02	0.61 (0.26-0.82)	.002	0.39 (0.21-0.55)	<.001
ICC	0.28 (−0.01 to 0.53)	.03	0.21 (−0.10 to 0.48)	.09	0.32 (−0.01 to 0.59)	.03	0.37 (0.16-0.54)	<.001	0.34 (0.05-0.59)	.01	0.46 (0.09-0.73)	.006	0.37 (0.19-0.53)	<.001

**<6 h (n = 79)^a^**	**1 (*n* = 36)**		**2 (*n* = 25)**		**3 (*n* = 18)**		**<0.5 (*n* = 47)**		**≥0.5 (*n* = 32)**		**0, 1, or 2a (*n* = 13)**		**2b, 2c, or 3 (*n* = 66)**	

Pearson	0.34 (0.01-0.60)	.04	0.27 (−0.14 to 0.60)	.19	0.35 (−0.14 to 0.70)	.15	0.33 (0.05-0.56)	.02	0.42 (0.08-0.67)	.02	0.82 (0.49-0.94)	<.001	0.33 (0.10-0.53)	.006
ICC	0,31 (−0.03 to 0.58)	.04	0.27 (−0.13 to 0.60)	.09	0.32 (−0.10 to 0.67)	.07	0.30 (0.01-0,54)	.02	0.37 (0.05-0.63)	.01	0.54 (−0.01 to 0.84)	.01	0.31 (0.09-0.51)	.003

**6**-**24h (*n* = 39)^a^**	**1 (*n* = 11)**		**2 (*n* = 17)**		**3 (*n* = 11)**		**<0.5 (*n* = 31)**		**≥0.5 (*n* = 8)**		**0, 1, or 2a (*n* = 10)**		**2b, 2c, or 3 (*n* = 29)**	

Pearson	0.09 (−0.54 to 0.66)	.78	−0.09 (−0.55 to 0.41)	.73	0.42 (−0.24 to 0.81)	.20	0.50 (0.18-0.73)	.004	0.07 (−0.67 to 0.74)	.86	0.35 (−0.36 to 0.80)	.32	0.57 (0.26-0.77)	.001
ICC	0.08 (−0.61 to 0.64)	.42	−0.09 (−0.55 to 0.41)	.63	0.39 (−0.30 to 0.79)	.12	0.46 (0.12-0.69)	.005	0.08 (−0.78 to 0.73)	.43	0.34 (−0.39 to 0.79)	.17	0.54 (0.23-0.75)	<.001

Abbreviations: ICC = intraclass correlation coefficient, HIR = hypoperfusion intensity ratio, TICI = thrombolysis in cerebral infarction.

a Time from last known well was unknown in three patients.

**Table 3.** Convolutional neural network estimated infarct volumes and final infarct volumes in mL, median (IQR), and corresponding *P*-values according to collateral status assessed with the Miteff scale or hypoperfusion intensity ratio (HIR) and according to outcome of thrombectomy assessed with the thrombolysis in cerebral infarction scale (TICI).

	Miteff				HIR			TICI		
All patients (*n* = 121)	1 (*n* = 47)	2 (*n* = 42)	3 (*n* = 32)	*P*-value	<0.5 (*n* = 81)	≥0.5 (*n* = 40)	*P*-value	0, 1, or 2a (*n* = 23)	2b, 2c, or 3 (*n* = 98)	*P*-value
CNN	80 (52-124)	37 (14-54)	26 (7-55)	<.001	38 (16-61)	91 (52-129)	<.001	52 (29-80)	46 (17-83)	.90
FIV	68 (13-125)	24 (4-45)	11 (4-27)	.002	17 (4-59)	39 (10-99)	.52	89 (15-125)	17 (5-54)	.06

**<6 h (*n* = 79)^a^**	**1 (*n* = 36)**	**2 (*n* = 25)**	**3 (*n* = 18)**		**<0.5 (*n* = 47)**	**≥0.5 (*n* = 32)**		**0, 1, or 2a (*n* = 13)**	**2b, 2c, or 3 (*n* = 66)**	

CNN	70 (43-109)	35 (12-67)	26 (6-50)	<.001	35 (14-63)	86 (50-133)	<.001	65 (33-76)	48 (14-89)	.96
FIV	45 (12-124)	19 (3-67)	10 (2-33)	.07	19 (4-71)	19 (7-98)	.89	110 (41-136)	14 (5-54)	.01

**6**-**24 h (*n* = 39)^a^**	**1 (*n* = 11)**	**2 (*n* = 17)**	**3 (*n* = 11)**		**<0.5 (*n* = 31)**	**≥0.5 (*n* = 8)**		**0, 1, or 2a (*n* = 10)**	**2b, 2c, or 3 (*n* = 29)**	

CNN	113 (80-130)	41 (23-46)	20 (2-63)	<.001	41 (20-59)	91 (67-129)	.04	47 (21-92)	44 (21-86)	.79
FIV	77 (40-165)	22 (6-40)	15 (4-17)	.002	15 (6-39)	81 (47-147)	.04	45 (12-99)	22 (8-50)	.79

Abbreviations: HIR = hypoperfusion intensity ratio, TICI = thrombolysis in cerebral infarction.

a Time from last known well was unknown in three patients.

## Results

Patient characteristics are presented in Table 1.

**Table 1.** Patient characteristics.

Number of patients	121
Age (years), mean (SD, range)	68 (12.3, 38-92)
Male sex, number (%)	64 (53)
NIHSS, median (IQR)^a^	13 (7-18)
Time from symptom onset to start CTA (min), median (IQR)^b^	240 (111-431)
Intravenous thrombolysis, number (%)	45 (37)
**Most proximal target occlusion location, *n* (%)**	
Distal ICA	12 (10)
MCA M1	77 (64)
MCA M2	32 (26)

Abbreviations: NIHSS = National institutes of health stroke scale, CTA = computed tomography angiography, MCA = middle cerebral artery.

a NIHSS was reported for 118 patients.

b Exact time from last known well was unknown in three patients.

When all patients were included in the analysis, the strength of correlation between CNN-derived volume estimates and FIV was moderate (*r *=* *0.44, *P* < .001). When all patients were classified according to collateral circulation as assessed with the Miteff system, only weak to moderate correlations were found as shown in Table 2. The same was also true when classification was based on HIR values. In patients presenting 6-24 hours after symptom onset, statistically significant moderate correlations were found in the HIR <0.5 and successful thrombectomy groups.

The strength of correlation between CNN-derived volume estimates and FIV was strong in patients with futile thrombectomy (*r *=* *0.61, *P* = .002) when all patients were included in the analysis. A strong correlation was also found in patients presenting <6 h from LKW (*r *=* *0.82, *P* < .001). This latter group was small (*n* = 12), however, and the CNN had a tendency for underestimating the FIV.

**Figure 3.**
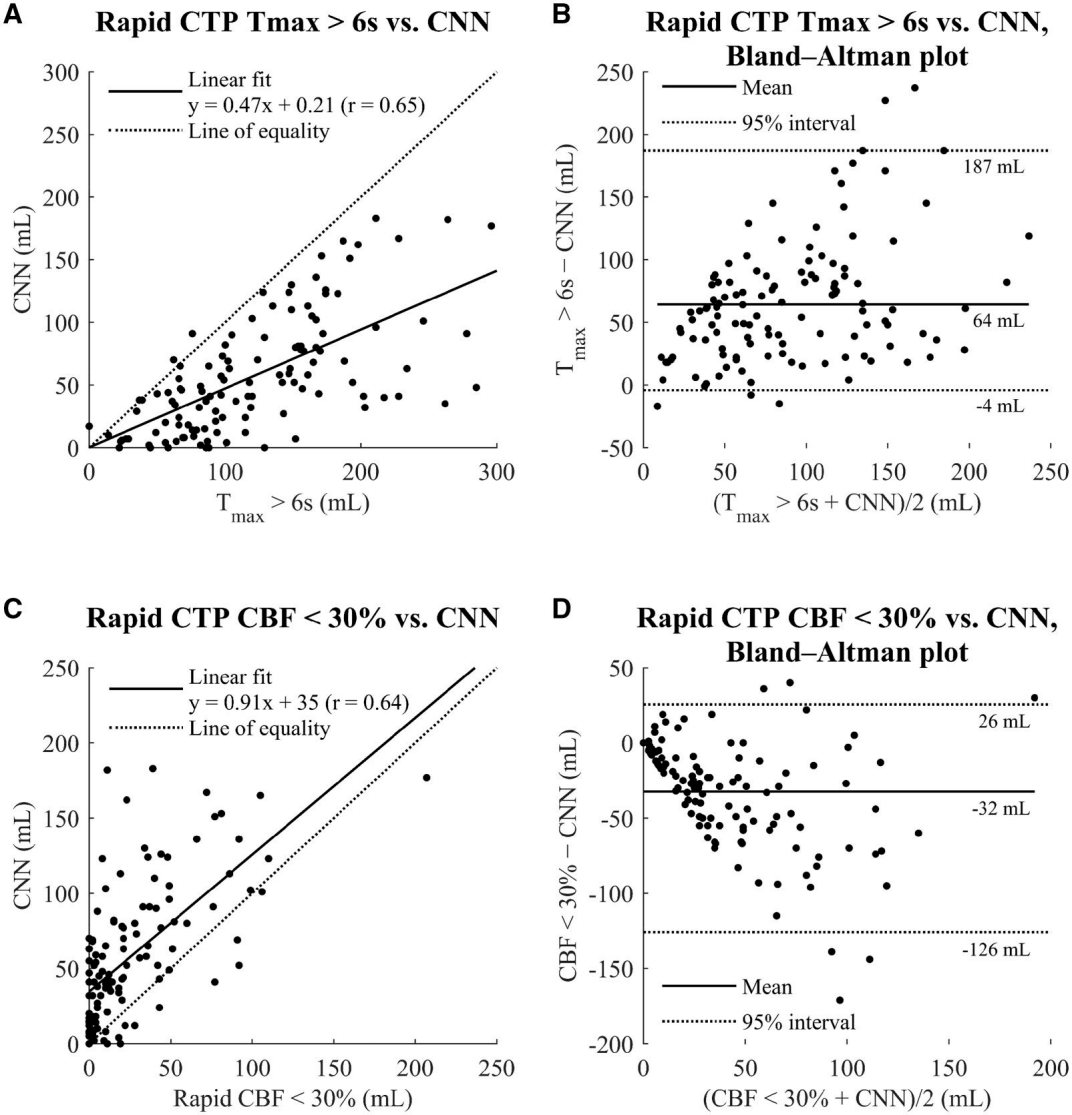
Correlation and Bland-Altman plots between CNN estimates and Rapid CTP penumbra (T_max_ > 6 s) (A, B) and core (CBF <30%) volumes (C, D). In the Bland-Altman plots, the solid lines show the mean differences, and the dotted lines represent 2.5th and 97.5th percentiles. Abbreviations: CNN = convolutional neural network, CTP = CT perfusion, T_max_ = time-to-maximum, CBF = cerebral blood flow.

Among all patients regardless of collateral status or thrombectomy result, a strong correlation between CNN estimates and Rapid CTP estimates of infarct core and penumbra was found, with the CNN showing a tendency to markedly underestimate the penumbra and to a lesser extent overestimate the infarct core as estimated by Rapid CTP (Figure 3).

The majority of patients (*n* = 104) had a Rapid CTP infarct core estimation of <50 mL. In this group, the correlation coefficient between CNN estimates and Rapid CTP core estimates (*r *=* *0.55, *y* = 1.50*x* +27.0) was comparable to the correlation in the entire study population, albeit with more severe overestimation. In contrast, among the 18 patients with Rapid CTP estimated infarct core volumes of >50 mL, correlation with CNN estimates was *r *=* *0.45 (*y* = 0.55*x* + 62.2).

When comparing all patients, a statistically significant difference was found in the medians of both the CNN estimates and FIV between patients with good or poor collaterals based on the Miteff system (Table 3).

This finding was also true for patients presenting in the 6-24-h time window. However, when patients were classified according to HIR-values, a statistically significant difference in FIVs could only be seen in the 6-24-h time frame. No difference was found in CNN estimates between successful and futile thrombectomies. In patients presenting <6 h from LKW, there was a significant difference in median FIV with notably smaller FIV in patients who underwent a successful thrombectomy.

When all patients were plotted on a time-attenuation curve, we found that one CTA was timed somewhere between the early arterial and mid-arterial phases, but all other 120 patients were imaged in the mid-arterial phase.

## Discussion

In this study, assessing factors that could possibly influence the reliability of a CTA-based CNN in infarct volume estimation, there was no significant difference in correlation coefficients between good and poor collateral circulation. The best correlation with FIV was found in patients that presented <6 h from symptom onset and who had a futile thrombectomy. Analysing the timing of CTA studies in relation to CTP time attenuation curves revealed that practically all patients were imaged in the mid-arterial phase. This could explain why our previous study^12^ found a better correlation between CNN estimates and FIV in patients who were treated with intravenous thrombolysis or supportive care only, as CNN estimates seem to include some penumbra in addition to the infarct core. This finding is also supported by evidence suggesting that infarct volume could evolve due to intracranial pressure changes occurring in the first day after stroke.^22^ However, regarding volumetric data, the CNN estimates fall somewhere between the Rapid CTP estimates of penumbra and core volumes (Figure 3). These findings are in line with previous studies, that have explored the effect of CTA timing on infarct core volume estimates.^16^^,^^23^^,^^24^ With older generation CT-scanners and slower image acquisition, CTA-based core volume estimations were found to be comparable to DWI.^6^ However, subsequent studies have shown contradictory results, which have been thought to be due to faster scanning times and the aim to optimize contrast opacification in cerebral arteries to facilitate LVO detection.^16^^,^^23^^,^^24^

In 2012, Pulli and Yoo emphasized that until protocol parameters are standardized, CTA should be used cautiously to inform treatment decisions in patients with AIS.^25^ Of particular interest is that in studies that reported good correlation with DWI or cerebral blood volume-based core volumes, image acquisition was slower than with modern scanners or delay time was based on peak enhancement at the superior sagittal sinus. In a recent study in this field, Estrada et al deduced that the optimal imaging phase for infarct core estimation on CTA is between the mid-arterial and late venous phase.^26^ This fits well with our results, as previous studies have shown that CTA overestimates the infarct core when faster scanning and delay times are used, which is due to the fact that a steady-state of contrast opacification in brain tissue has not yet been achieved.

Previous studies from other groups using CTA-based automated or machine learning methods for infarct volume estimation have not addressed the issue of CTA timing. Reidler et al made a notion of CTA timing though, to explain their finding that their method based on relative HU-values overestimated infarct core volumes and FIV with respective correlation coefficients of *r *=* *0.29 and *r *=* *0.32.^10^ Wang et al on the other hand achieved good correlation with Rapid CTP core estimations using an additional CTA acquisition with an 8 s additional delay after standard CTA acquisition.^11^ Sheth et al did not disclose details of their CTA protocol, but it would be reasonable to assume that it was similar to ours, given that CNN correlation with Rapid CTP estimates of infarct core volumes was of the same magnitude as that achieved by their DeepSymNet (*r *=* *0.64 and *r *=* *0.70, respectively) and their data were gathered from patients presenting between 2016 and 2018.^8^

Our finding that the correlation between CNN predictions and FIV did not significantly change between good and poor collateral circulation can be thought of as a consequence of the early imaging phase as slower collateral flow has not yet reached the ischaemic area. Different results might have been attained had the CTA acquisitions been performed between the mid-arterial and delayed venous phases.

The fact that the CNN only achieved moderate correlation with FIV can also be related to the significant variation of FIV, as the median CNN volume estimates and FIVs still showed statistically significant differences between good and poor collaterals in a logical direction, that is, patients with good collaterals having both smaller CNN estimates and FIV. The same trend was also observed between successful and futile thrombectomies, with smaller median FIV in patients with successful thrombectomies, although a statistically significant difference was only observed in patients presenting <6 h from LKW. Finally, infarct evolution is a complex process that is affected by a myriad of factors, which leads to considerable uncertainty in FIV estimation regardless of the method used, be it conventional CTP^27^ or a deep learning-based method. Factors affecting the FIV estimations of our CNN have been explored in depth in a previous publication.^13^

The main limitation of this study was that due to the standardized CTA protocol in our institution, we found that practically all CTA acquisitions were made in the mid-arterial phase, which made it impossible to analyse the effect of CTA timing on CNN volume estimates in this study population. Instead, only suggestive interpretations regarding this variable could be made. A major limitation was also the use of a single observer for the segmentation of FIVs, which we aimed to address by double reading equivocal cases and using CTP maps and, when available, more recent CT studies for additional information. However, inter-observer variability has been shown to be low when interpreting follow-up CT studies for ischaemic stroke.^28^ The single-centre retrospective design of this study was also a limitation, which we aimed to address by selecting consecutive patients to simulate real-life performance. In the majority of cases, NCCT was used for follow-up, because a 24 h follow-up CT is the standard protocol in our institution. This limits the sensitivity of infarct detection somewhat compared to DWI. Different vendors were used for CTA imaging. However, one specific scanner was used in most studies, which limits the generalizability of the results. Likewise, biases in the original CNN training can affect the accuracy and repeatability of the results. Potential limitations include noise in the training data (such as annotator bias), the representativeness of patients in the original training data, and limited variability in scanner models and manufacturers.

In conclusion, our study showed no significant difference in the correlation of CNN-derived infarct volume estimates and FIV between good or poor collaterals or successful or futile thrombectomy. CTA acquisition was made in the mid-arterial phase in practically all patients in this study population, which is not ideal for core volume estimation and may help explain the limited correlation between CNN estimates and FIV found in this and previous studies. It would be crucial to optimise CTA timing in the future development of CTA-based deep learning methods to achieve the best results.

## References

[1] Powers WJ , Rabinstein AA, Ackerson T, et al Guidelines for the early management of patients with acute ischemic stroke: 2019 update to the 2018 guidelines for the early management of acute ischemic stroke a guideline for healthcare professionals from the American Heart Association/American Stroke Association. Stroke. 2019;50(12):e344-e418. 10.1161/str.0000000000000211 31662037

[2] Campbell B , Ma H, Ringleb PA, et al Extending thrombolysis to 4.5–9 h and wake-up stroke using perfusion imaging: a systematic review and meta-analysis of individual patient data. Lancet. 2019;394(10193):139-147. 10.1016/S0140-6736(19)31053-0 31128925

[3] Nogueira RG , Jadhav AP, Haussen DC, et al Thrombectomy 6 to 24 hours after stroke with a mismatch between deficit and infarct. N Engl J Med. 2018;378(1):11-21. 10.1056/NEJMoa1706442 29129157

[4] Turc G , Bhogal P, Fischer U, et al European Stroke Organisation (ESO) – European Society for Minimally Invasive Neurological Therapy (ESMINT) guidelines on mechanical thrombectomy in acute ischaemic stroke endorsed by stroke alliance for Europe (SAFE). Eur Stroke J. 2019;4(1):6-12. 10.1177/2396987319832140 31165090 PMC6533858

[5] Kim Y , Lee S, Abdelkhaleq R, et al Utilization and availability of advanced imaging in patients with acute ischemic stroke. Circ Cardiovasc Qual Outcomes. 2021;14(4):e006989. 10.1161/circoutcomes.120.006989 33757311

[6] Schramm P , Schellinger PD, Fiebach JB, et al Comparison of CT and CT angiography source images with diffusion-weighted imaging in patients with acute stroke within 6 hours after onset. Stroke. 2002;33(10):2426-2432. 10.1161/01.str.0000032244.03134.37 12364733

[7] Öman O, Mäkelä T, Salli E, Savolainen S, Kangasniemi M. 3D convolutional neural networks applied to CT angiography in the detection of acute ischemic stroke. *Eur Rad Exp*. 2019;3(1):8. 10.1186/s41747-019-0085-6 PMC6374492 30758694

[8] Sheth SA , Lopez-Rivera V, Barman A, et al Machine learning-enabled automated determination of acute ischemic core from computed tomography angiography. Stroke. 2019;50(11):3093-3100. 10.1161/strokeaha.119.026189 31547796

[9] Hilbert A , Ramos LA, van Os HJ, et al Data-efficient deep learning of radiological image data for outcome prediction after endovascular treatment of patients with acute ischemic stroke. Comput Biol Med. 2019;115:103516. 10.1016/j.compbiomed.2019.103516 31707199

[10] Reidler P , Puhr-Westerheide D, Rotkopf L, et al Cerebral attenuation on single-phase CT angiography source images: automated ischemia detection and morphologic outcome prediction after thrombectomy in patients with ischemic stroke. PLoS One. 2020;15(8):e0236956. 10.1371/journal.pone.0236956 32790766 PMC7425881

[11] Wang C , Shi Z, Yang M, et al Deep learning-based identification of acute ischemic core and deficit from non-contrast CT and CTA. J Cereb Blood Flow Metab. 2021;41(11):3028-3038. 10.1177/0271678X211023660 34102912 PMC8756471

[12] Hokkinen L, Mäkelä T, Savolainen S, Kangasniemi M. Evaluation of a CTA-based convolutional neural network for infarct volume prediction in anterior cerebral circulation ischaemic stroke. Eur Rad Exp. 2021;5(1):25. 10.1186/s41747-021-00225-1 PMC8222495 34164743

[13] Hokkinen L , Mäkelä T, Savolainen S, Kangasniemi M. Computed tomography angiography-based deep learning method for treatment selection and infarct volume prediction in anterior cerebral circulation large vessel occlusion. Acta Radiol Open. 2021;10(11):20584601211060347. 10.1177/20584601211060347 34868662 PMC8637731

[14] Elijovich L , Goyal N, Mainali S, et al CTA collateral score predicts infarct volume and clinical outcome after endovascular therapy for acute ischemic stroke: a retrospective chart review. J Neurointerv Surg. 2016;8(6):559-562. 10.1136/neurintsurg-2015-011731 25994937

[15] Regenhardt RW , González RG, He J, Lev MH, Singhal AB. Symmetric CTA collaterals identify patients with slow-progressing stroke likely to benefit from late thrombectomy. Radiology. 2022;302(2):400-407. 10.1148/radiol.2021210455 34726532 PMC8792270

[16] Mukherjee A , Muthusami P, Mohimen A, et al Noncontrast computed tomography versus computed tomography angiography source images for predicting final infarct size in anterior circulation acute ischemic stroke: a prospective cohort study. J Stroke Cerebrovasc Dis. 2017;26(2):339-346. 10.1016/j.jstrokecerebrovasdis.2016.09.026 27789149

[17] Marcus DS , Olsen TR, Ramaratnam M, Buckner RL. The extensible neuroimaging archive toolkit: an informatics platform for managing, exploring, and sharing neuroimaging data. Neuroinformatics. 2007;5(1):11-34. 10.1385/ni:5:1:11 17426351

[18] Fedorov A , Beichel R, Kalpathy-Cramer J, et al 3D slicer as an image computing platform for the quantitative imaging network. Magn Reson Imaging. 2012;30(9):1323-1341. 10.1016/j.mri.2012.05.001 22770690 PMC3466397

[19] Miteff F , Levi CR, Bateman GA, Spratt N, McElduff P, Parsons MW. The independent predictive utility of computed tomography angiographic collateral status in acute ischaemic stroke. Brain. 2009;132(Pt 8):2231-2238. 10.1093/brain/awp155 19509116

[20] Mäkelä T , Öman O, Hokkinen L, et al Automatic CT Angiography Lesion Segmentation Compared to CT Perfusion in Ischemic Stroke Detection: a Feasibility Study. J Digit Imaging. 2022;35(3):551-563. 10.1007/s10278-022-00611-035211838 35211838 PMC9156593

[21] Johnson HJ , Harris G, Williams K. BRAINSFit: mutual information rigid registrations of whole-brain 3D images, using the insight toolkit. Insight J. 57(1):1-10. 10.54294/hmb052

[22] Beard DJ , Murtha LA, McLeod DD, Spratt NJ. Intracranial pressure and collateral blood flow. Stroke. 2016;47(6):1695-1700. 10.1161/STROKEAHA.115.011147 26786117

[23] Sharma M , Fox AJ, Symons S, Jairath A, Aviv RI. CT angiographic source images: flow- or volume weighted? AJNR Am J Neuroradiol. 2011;32(2):359-364. 10.3174/ajnr.A2282 21051518 PMC7965723

[24] Yoo AJ , Hu R, Hakimelahi R, et al CT angiography source images acquired with a fast-acquisition protocol overestimate infarct core on diffusion weighted images in acute ischemic stroke. J Neuroimaging. 2012;22(4):329-335. 10.1111/j.1552-6569.2011.00627.x 21848682 PMC3248622

[25] Pulli B , Yoo A. CT angiography source images with modern multisection CT scanners: delay time from contrast injection to imaging determines correlation with infarct core. AJNR Am J Neuroradiol. 2012;33(4):E61. 10.3174/ajnr.A3039 22322617 PMC8050457

[26] Estrada UM , Meeks G, Salazar-Marioni S, et al Quantification of infarct core signal using CT imaging in acute ischemic stroke. Neuroimage Clin. 2022;34:102998. 10.1016/j.nicl.2022.102998 35378498 PMC8980621

[27] Goyal M , Ospel JM, Menon B, et al Challenging the ischemic core concept in acute ischemic stroke imaging. Stroke. 2020;51(10):3147-3155. 10.1161/STROKEAHA.120.030620 32933417

[28] Boers AM , Marquering HA, Jochem JJ, et al MR CLEAN Investigators Automated cerebral infarct volume measurement in follow-up noncontrast CT scans of patients with acute ischemic stroke. AJNR Am J Neuroradiol. 2013;34(8):1522-1527. 10.3174/ajnr.A3463 23471018 PMC8051473

